# ‘I took care of my kids’: mothering while incarcerated

**DOI:** 10.1186/s40352-020-00109-3

**Published:** 2020-06-05

**Authors:** Stephanie C. Kennedy, Annelise M. Mennicke, Chelsea Allen

**Affiliations:** 1 Institute for Justice Research and Development, College of Social Work, Florida State University, 2010 Levy Ave, Suite 3400, Tallahassee, FL 32310 USA; 2 School of Social Work, University of North Carolina at Charlotte, Charlotte, NC 28223 USA; 3 Graduate School of Social Work, Columbia University, New York, NY 10027 USA

**Keywords:** Incarcerated women, Mothers, Mothering, Maternal incarceration, Mental health, Substance use disorders, Gendered punishment, Qualitative methods

## Abstract

**Background:**

Little is known about how incarcerated mothers make meaning of their parenting role and relationship with their children prior to incarceration and during custody. The aims of this project were to explore the experiences of mothering prior to incarceration and during custody using the Gendered Pathways Perspective and to examine how mothering intersects with incarcerated women’s health and health outcomes to facilitate prevention and intervention strategies. This secondary data analysis used qualitative methods and grounded theory to identify themes related to mothering from 41 incarcerated mothers. Analyses were conducted by two independent coders, each of whom interviewed women as part of the primary study.

**Results:**

Identified themes highlight how mothers sacrificed their own health and wellness in order to parent their children, sometimes foregoing substance use disorder treatment because they had no childcare options. Additionally, incarcerated mothers described the psychological distress of family separation and asked for additional parenting programs to increase mother-child connection. Finally, mothers suggested that capitalizing on the mothering role might be a potent mechanism for change, especially as related to substance use disorder treatment.

**Conclusions:**

Research on incarcerated parents often focuses on their children, which obscures incarcerated mothers’ needs related to health and wellness. The prison environment offers few opportunities to foster mother-child connection; most mothers never receive even one visit from their children. Incarcerated mothers contextually framed crime as protecting and providing for children and identified community-based and in-prison service gaps. Recommendations include infusing mothering and caretaking responsibilities into the sentencing process and exploring the intersection of race, gender, class, and mothering status on criminalized behavior. Additionally, there is an urgent need to expand the availability of community-based and in-prison programs that allow women to address health concerns while mothering their children.

## Introduction

Currently, more than 225,000 women are behind bars in jails and prisons across the United States, and a million more are under some form of correctional supervision (e.g., probation, parole, or community supervision; Bronson & Carson, 2019; Kaeble, 2018; Zeng, 2019). The vast majority of these women are mothers – more than 80% of incarcerated women have at least one child (Swavola, Riley, & Subramanian, 2016). When compared to incarcerated fathers, incarcerated mothers are far more likely to have primary or sole custody of their children, and to have had at least one minor child living in their home at the time of their arrest (Glaze & Maruschak, 2008). Relatively little research documents the experiences of parenting while incarcerated, especially from the perspective of the mother. Further, how incarcerated mothers’ parenting intersects with health and well-being is unclear. The purpose of this project was to explore the experiences of mothering before and during incarceration using women’s own words and to examine how mothering intersects with incarcerated mothers’ health and health outcomes. The aims of this project were to amplify the voices of incarcerated mothers and generate prevention and intervention policies and practices to improve the health and well-being of incarcerated women and their children and families.

## Background

### Gendered pathways perspective

The Gendered Pathways Perspective (GPP) emerged in the last decades of the twentieth century as a framework for understanding women’s intersection with both crime and the criminal justice system (e.g., Daly, 1992; Owen, 1998; Richie, 2018). The GPP explores the social and psychological realities unique to the female experience and identifies women’s pathways into the criminal justice system. Results from GPP research suggest that women engage in criminalized behavior based on factors: (a) Not typically seen among men (e.g., prostitution, intimate partner violence, and coercion); (b) More prevalent among women (e.g., sexual abuse); or (c) Common among men and women but have distinctly gendered effects for women (e.g., drug use, intimate relationships, poverty, and economic marginalization; Belknap & Holsinger, 2006; Bloom, Owen, & Covington, 2003; Daly, 1992; Reisig, Holtfreter, & Morash, 2006). These factors underscore women’s criminalized behavior as a function of surviving both victimization and poverty and indicate that women differ from men in the context of their criminalized behaviors (Owen, 1998; Richie, 2001, 2018; Stark, 2007). Few studies employing the GPP lens, however, have specifically investigated motherhood and mothering as a potential pathway to criminalized behavior (Parry, 2018). Motherhood creates additional layers of constraint and opportunity for vulnerable women prior to incarceration and during custody. Therefore, in the current analysis, we expand the GPP theoretical frame to investigate how incarcerated women’s experience of mothering influences their health outcomes and creates a potent pathway to prison for this vulnerable population.

### Incarcerated mothers

Although most incarcerated parents are men – more than 1.1 million fathers are incarcerated in state and federal prisons compared to 150,000 mothers – women’s incarceration is far more disruptive to families than men’s incarceration (e.g., Smyth, 2012). Incarcerated women are significantly more likely to be the sole or custodial parent compared to incarcerated men – 64% of women compared to 47% of men (Glaze & Maruschak, 2008). Further, although incarcerated fathers indicate that the overwhelming majority of their children (in excess of 90%) live with their mothers while the father is in prison, incarcerated mothers describe a complex web of formal, informal, and state-appointed caretakers. More than half of incarcerated women’s children live with a grandparent; only a quarter live with their fathers during the mother’s custody (Johnson & Waldfogel, 2004). The children of incarcerated mothers are eight times more likely to be placed in foster care and seven times more likely to be placed in a group home or institutional setting when compared to the children of incarcerated fathers (Dallaire, 2007). The incarceration of mothers, therefore, has a profoundly destabilizing effect on both children and families, as evidenced by the wealth of literature exploring negative outcomes for the children of incarcerated mothers (e.g., Aiello & McKorkel, 2018; Dallaire, Zeman, & Thrash, 2015; Huebner & Gustafson, 2007).

The needs of incarcerated mothers, however, have received far less scholarly attention than the needs or struggles of their children. It is well known that the overwhelming majority – in excess of 75% – of incarcerated women report experiences of physical and sexual abuse, bullying, peer victimization, and witnessing violence in childhood (e.g., Asberg & Renk, 2013; DeHart, 2008, 2009; Kennedy et al., 2016; Messina & Grella, 2006; Salisbury & Van Voorhis, 2009; Tripodi et al., 2019; Wolff et al., 2009). When interviewed, incarcerated women often link their criminalized behavior directly to coping with their experiences of abuse (e.g., drug crimes), economic deprivation caused by poverty and child caretaking responsibilities (e.g., property crimes and fraud; DeHart, 2008; DeHart, Lynch, Belknap, Dass-Brailsford, & Green, 2014; Fuentes, 2014; Grella & Greenwell, 2006; Kennedy & Mennicke, 2018; Lynch, Dehart, Belknap, & Green, 2012), and surviving domestic abuse (e.g., violent crime; Pollack et al., 2006). Studies that focus on incarcerated mothers suggest that mothers report higher rates of child maltreatment when compared to incarcerated fathers (Allen, Flaherty, & Ely, 2010; Casey-Acevedo, Bakken, & Karle, 2004) and mothers are far more likely than men to experience domestic violence and to come to prison through intimate partner entanglements (e.g., Barlow, 2016; Richie, 2001). These entanglements may include being coerced to use or sell drugs, forced to engage in prostitution, or may have resulted in women murdering their abusive partner (DeHart et al., 2014; Fedock, 2018; Pollack et al., 2006; Stark, 2007). Some incarcerated mothers described how pregnancy and motherhood complicates existing troubling relationships with abusive intimate partners, creating a nearly inescapable cycle of violence (e.g., DeHart, 2008; Fuentes, 2014).

### Mothering while incarcerated

Comparative gender analyses suggest that women report an acutely more painful experience of confinement when compared to men and that their psychological well-being and mental health are compromised by imprisonment (Crewe et al., 2017; Harner & Riley, 2013). Psychological and emotional distress are amplified for incarcerated mothers, as prisons were not designed to manage the needs of mothers and their young children (e.g., Wattanaporn & Holtfreter, 2014). For many incarcerated mothers, family relationships are effectively severed during incarceration (Aiello & McCorkel, 2018). The process of incarceration, prison visitation policies, and lack of intensive family-oriented programming further fractures the mother-child bond and exacerbate psychological distress among incarcerated mothers (The Rebecca Project for Human Rights, 2010). Although there are proven benefits to both mothers and their children through regular contact (e.g., Poehlmann, 2005a, 2005b), most mothers never receive even one visit from their children during their incarceration (Glaze & Maruschak, 2008; Mignon & Ransford, 2012). Some mothers report not wanting their children or loved ones to see them in the prison setting, many more mothers identify the insurmountable logistical barriers which prevented visits (e.g., Allen et al., 2010). For example, as there are simply fewer women’s prisons than men’s prisons, women are incarcerated, on average, approximately 160 miles away from home (Travis, McBride, & Solomon, 2005). The prison environment also presents specific obstacles to mother-child visitation such as inadequate information about the visitation process, difficulty scheduling visits, uncomfortable or humiliating visitation processes, or the family’s inability to access or afford transportation.

Visitation is further complicated by many incarcerated mothers’ dependence on their children’s caregivers. One of the most significant obstacles to mothers receiving visits from their children are caretakers or foster parents who are unwilling to facilitate visits to the prison, citing both logistical barriers and emotional concerns (e.g., not wanting to upset the child/ren; Travis et al., 2005). The physical distance, paired with economic deprivation and the complexity of caretaking relationships for the children of incarcerated mothers, means that very few mothers receive regular visits from their children. For mothers who do receive visits, frequent and flexible communication with children is associated with decreased emotional and psychological distress, as well as decreased parenting stress (Aiello, 2016; Houck & Loper, 2002; Stringer & Barnes, 2012).

Continued contact during incarceration eases the psychological stress of separation for both mother and child, and is associated with more responsible parenting, increased motivation for change, and more secure attachment and bonding (Mignon & Ransford, 2012; Schubert, Duininck, & Shlafer, 2016). Likewise, visits help ease anxiety for mothers preparing for release (Mancini et al., 2016). On the other hand, infrequent visitation strains the mother-child relationship and is linked to in-prison behavior infractions, which, in turn, directly result in the suspension of visitation privileges and, in some cases, the termination of one’s eligibility for visitation programs altogether (Casey-Acevedo et al., 2004). Interviews suggest that many incarcerated mothers have attachment disorders and struggle to find security in their emotional bonds with their own children. Reducing or eliminating contact between mothers and children exacerbates this insecurity, making reconnection upon release from incarceration far more difficult.

### Prison parenting programs

While correctional policies and procedures are beginning to conceptualize incarcerated mothers and pregnant women in prison as vulnerable populations, policy implementation is erratic and family-oriented programs are rarely available to all eligible women (e.g., The Rebecca Project for Human Rights, 2010). For example, as of 2018, only 22 states had passed laws prohibiting the shackling of pregnant women during labor and birth (Ferszt, Palmer, & McGrane, 2018). While didactic parenting classes are available in more than 90% of women’s correctional facilities (Pollock, 2003), these programs typically focus on child development and were not designed to mediate the psychological distress inherent to family separation for many incarcerated mothers. Promising gender-responsive and trauma-informed programs are beginning to be implemented within the prison setting (e.g., Tripodi, Mennicke, McCarter, & Ropes, 2017), although these programs center on experiences of posttraumatic stress and substance use, and do not engage women as mothers or integrate mothering comprehensively into intervention content. While it is certainly important to acknowledge that not all incarcerated women are mothers and not all mothers value the mothering identity, the mothering role is an underutilized potential mechanism for health-oriented change for those women who do value mothering (Thompson & Harm, 2000).

Few prison nursery programs are available to incarcerated mothers nationally – only eight states have any prison nursery program, often run out of only one women’s prison (Carlson, 2018). These programs typically allow infants to co-reside with their mothers in a segregated unit until they are 3 to l8 months old. In general, eligible mothers must be serving sentences for non-violent offenses and their children must be born during the mother’s incarceration (Women’s Prison Association, 2009). Programs serve between 5 to 29 mother-child pairs and have been shown to improve mother-child attachment, improve parenting efficacy, and reduce participant recidivism rates (Fritz & Whiteacre, 2016). Importantly, these programs are also associated with decreased psychological distress for mothers (Luther & Gregson, 2011). In cases where no prison nursery program is available, women who give birth during incarceration are separated from their newborn within 24 to 72 h (The Rebecca Project for Human Rights, 2010).

Some states offer more intensive parenting programs to incarcerated mothers who meet eligibility criteria, although it is unclear how many such programs exist as they are rarely run by the department of corrections. For example, incarcerated mothers in one prison in North Carolina are able to visit with their children on prison grounds in a home-like visitation center (Mothers and their Children - MATCH, n.d..). This non-profit organization provides visitation services, parenting education and support, and financial assistance for families to travel for visits. However, although comprehensive support services are offered to mothers and caregivers, the organization focuses explicitly on improving the psychological development of the child. While the opportunity they provide to mothers is laudable, few – if any – programs exist that intentionally foreground the needs of incarcerated mothers.

Unfortunately, there is emerging evidence to suggest that the tension between rehabilitation (in the form of gender-responsive and trauma-informed programs) and punishment (the penal paradigm) may not be able to be reconciled in locked spaces (e.g., Aiello, 2013; Belknap, Lynch, & DeHart, 2016). When surveyed, few correctional administrators are familiar with more intensive parenting programs or prison nurseries (Campbell & Carlson, 2012). Even in prison nurseries, incarcerated mothers are palpably aware of the tensions that arise in their enactment of the roles of both ‘mother’ and ‘inmate’ (Luther & Gregson, 2011). The correctional environment is designed to control all aspects of the lives of incarcerated individuals – incarcerated mothers note how the prison milieu limits their decision-making power as mothers and stymies their ability to create safety and a home-like environment for themselves and their children (Aiello, 2013, 2016; Luther & Gregson, 2011).

### Existing gaps

The perspectives of incarcerated women, and especially of incarcerated mothers, are rarely reflected in research, policy, or intervention content. Typically, the focus is on the needs of children of incarcerated parents (e.g., Aiello & Mccorkel, 2018; Dallaire et al., 2015; Huebner & Gustafson, 2007) or the identification and treatment of mental health, substance use disorder, or physical health deficits of incarcerated women more broadly (e.g., Kennedy et al., 2016; Messina & Grella, 2006). Few scholars explore how incarcerated mothers conceptualize their needs prior to incarceration and during custody and examine which supports mothers feel will increase success and improve well-being after they are released from incarceration. It is vital that we learn more about the experiences and needs of incarcerated mothers as a means to develop more effective physical, mental, and behavioral health prevention and intervention strategies, foster the parent-child bond between mothers and their children, and help set women and families up for success when they return home.

Incarcerated mothers’ own voices have often been overlooked when identifying strategies to reform the prison environment or generate content for intervention development; more research is needed to use the voices of incarcerated mothers to guide policy and program design. The purpose of the current study is to explore the experience of motherhood for incarcerated women using the Gendered Pathways Perspective and qualitative interviews. Specifically, we aim to understand the health impacts of mothering prior to incarceration and during custody as a means to incorporate mothering into prevention and intervention efforts to improve their health and well-being and ensure the healthy development of their children and families.

## Method

### Procedures

The current project analyzed qualitative data collected for a larger study which evaluated the relationship between childhood abuse and behavioral health outcomes among incarcerated women. Women were recruited from three state prisons in the southeastern US; the sample was randomly selected using the census of all women housed in a minimum/medium supervision prison in Florida (*n* = 39), a minimum security prison in North Carolina (*n* = 74), and a medium/close supervision prison in North Carolina (*n* = 74). Data collection occurred from June 2015 to July 2017. All procedures were approved by the [university removed for review] and the [university removed for review], and the Department of Corrections Human Subjects Review Boards in Florida and North Carolina. To be eligible, participants had to be at least 18 years old, English-speaking, indicate that they understood the nature of the study and what being a participant entailed, and provide informed consent. All participants were interviewed by a research team member who read items out loud and recorded participant responses. Interviews were conducted in a large common space like a visitation room or classroom; correctional officers were not present for interviews. Overall, 306 women were randomly selected for recruitment and 187 women joined the study, representing a 61% response rate.

### Participants

The current secondary data analysis was conducted using the qualitative responses of 41 of the 187 women, as these 41 women described some facet of mothering or parenting in their qualitative responses. On average, the 41 mothers in the sample were 38 years old (SD = 10.9; range: 23–63) and self-identified as White (67%), Black (25%), and Native American (8%). The mean sentence length was 5.9 years (SD = 7.2 years), with a range of 90 days to 38 years. Additionally, eight mothers (20%) were serving at least one life sentence, with three mothers reporting more than a life sentence (e.g., multiple life sentences, or a life sentence plus additional years). Current charges were most often related to violent (54%), property (31%), and drug crimes (15%). One participant chose not to disclose her current charges. Data on family composition and the number and ages of each mother’s child/ren were not collected in the primary study.

### Data collection tool

Each participant was interviewed by a member of the research team who was a social worker with clinical interviewing experience. After completing a structured interview, women were asked two open-ended questions about how their childhood experiences affected their life trajectory and how we could better help women like them. The interviewer recorded her answer using brief, direct quotes, writing down the participant’s words exactly as they were spoken. When the participant had finished responding to the prompt, the interviewer read the comments back to her, allowing her an opportunity to edit, alter, add to, or rescind any comments. Although this documentation method was far from ideal, there are anonymity and confidentiality risks associated with using recording devices in prison; therefore, we decided to introduce systematic error into the data via recording procedures to ensure study participants’ rights were protected. As there were no specific prompts in the primary study about mothering/parenting, the themes explored in this analysis emerged organically.

### Data analysis

The current analysis was conducted by two of the primary study researchers; together these researchers conducted the majority of the 187 interviews. These cis-gender women identified as White, mostly heterosexual, and middle-class. Data were deidentified and entries were read multiple times by each coder prior to starting the coding process. Then, data were engaged in a line-by-line, case-by-case fashion. Verbatim quotes transcribed from the brief interview were analyzed using a grounded theory approach involving an inductive, iterative process of coding and memoing (Charmaz, 2006). After the 41 relevant entries were identified, data were broken up into component parts or properties, and codes were developed by each coder independently to reflect the content of data. Memos were exchanged to suggest emerging themes and to examine the boundaries of consensus. Mothering was a prominent theme in the data, despite the fact that examining mothering or parenting among incarcerated women was not the purpose of the initial project. Consensus was achieved about both the codes and the themes they represented. Finally, incarcerated mothers’ discussions of motherhood and mothering were synthesized and presented in dominant themes. A decision was made to refer to the mothers in the sample by participant number rather than by pseudonyms as the sample was quite large for a qualitative analysis and we were concerned that our choice of pseudonym (without participant input) would add an unnecessary layer of bias for readers. Additionally, we report the race, current charges, and relevant criminal justice system history for each mother identified. We chose not to report participant age to ensure anonymity for each mother.

## Results

Women described the intersection of psychological distress, criminalized behavior, and mothering prior to incarceration and they were palpably aware of having made choices to sacrifice their own health on behalf of their children. Mothers also discussed the lack of family services during custody and their distress at losing both the physical and emotional connections with their children due to family separation and the general lack of available comprehensive visitation programs. Women underscored how their identities as mothers could be used to catalyze their own change processes. Each of these themes are explored below.

### Psychological distress and criminalized behavior prior to incarceration

The mothers we interviewed noted that their decision-making processes were often guided by their roles as mothers and the primacy of their mothering identities. Although many of the women in the sample had become embroiled in the criminal justice system prior to becoming mothers, they noted being viewed as independent and disconnected from their children after becoming incarcerated. For example, participant 7, a White mother, was serving 13 months for a probation violation on her original charge of possession of a controlled substance without a prescription. She noted, “I feel guilt about ending up here. I feel like as soon as I had a daughter I should have been more responsible.” She asked for family counseling to help heal these wounds, saying that she needed “One-on-one counseling for me and my daughter. To better help me, help my 14-year-old daughter.”

Other mothers discussed having made a range of decisions, including illegal ones, on behalf of their children. Mothers connected their crime to experiences of trauma, identifying how they were forced into criminalized behavior to survive and cope with that survival (e.g., Kennedy & Mennicke, 2018). Germane to the current analysis was that mothers’ stories of survival demonstrated how they foregrounded the well-being of their children, in striking contrast to dominant societal narratives which frame incarcerated mothers as selfish and thoughtless (e.g., Aiello, 2016; Allen et al., 2010). Mothers spoke of how they prioritized their children, even when that meant risking their own autonomy and freedoms. For example, participant 1, a White mother, was serving her third adult incarceration for drug crimes. She mused about choices she had made to protect her children from their abusive father and said, “When they [mothers] aren’t getting help, they gotta do what they have to do to protect their children.” She had been arrested and incarcerated for the first time at age 12 for arson, which she described as “trying to burn my house down with my step-dad in it because he was very abusive.” This phrase – that as a mom ‘you gotta do what you gotta do’ was woven throughout mothers’ responses. In this vein, participant 11, a Native American mother, talked about how she had “taken charges” for a 13-year-old son to keep him out of the system. “I knew it would be easier and faster for me to be here than to risk him losing everything,” she said.

Mothers also framed their engagement in other criminalized behaviors, even violent crime, in the context of mothering. For example, participant 9, a Black mother, stated, “I had to be aggressive in the streets to take care of my kids…. I worked. I took care of my children. But I had to be aggressive to take care of us.” This participant was serving 30 months for battery on a law enforcement officer, her second adult incarceration for starting a fight in the community and continuing the fight when law enforcement arrived. She had been first arrested at age 15 for fighting on school grounds. Protection and care, for some mothers, extended beyond providing food and shelter, and included ensuring that children were physically safe in their environments.

In some cases, this need to protect their children pushed mothers to violence or extreme behavior. For example, participant 49, a White woman, detailed her marriage to an extremely violent man. After he threatened to harm her children, she borrowed a gun from a neighbor and tried to kill him. She spoke of waiting until he fell asleep and sitting in the darkness with the gun aimed at his head. Unable to pull the trigger, she hired someone to kill him for her. She said at the conclusion of her story, “I didn’t want my daughter to be scared, I didn’t want him to hit me anymore.” She was incarcerated at age 30 and will spend the rest of her natural life in prison for capital murder.

What was apparent in these narratives was that the “decision” to engage in criminalized behavior was far more layered and complex than is typically presented in the media or in common conceptions of women’s motivation to “do crime.” Far from irresponsible or neglectful, the mothers we interviewed told stories of engaging in illegal activities because of, not despite, their children. Their reactions were often fueled by psychological distress of having survived abuse and extraordinary trauma. For example, participant 58, a Black mother, recounted the horrific story of becoming an accomplice to murder. She drove her boyfriend and their infant child to a store; her boyfriend entered the store alone, then robbed and murdered the employees. He returned to the car and screamed at her to drive. When she hesitated, he told her that he would “gut the baby from head to toe” if she stopped driving. With no viable options to ensure survival for herself or her baby, she drove the car as instructed. She was serving three life sentences for conspiracy to commit murder and will never be a part of her child’s life.

Although the authors, as well as most of the mothers we interviewed, acknowledged that many of their decisions were far from ideal, the context of women’s criminal offending was illuminating. In many cases, mothers were trapped between two terrifying decisions, and they were aware that both “choices” would lead directly to terrible outcomes. However, mothers described that they felt compelled to act because they connected the pain of not acting to either dying or watching as their children were hurt – physically or emotionally.

### Sacrificing health on behalf of children prior to incarceration

Foregoing help-seeking behaviors in order to care for children was included as a component of the mothers’ decision-making prior to incarceration. Mothers noted how existing services were not accessible or available to women who needed childcare or residential treatment. This gap existed across service spectrums, including mental health treatment, substance use disorder treatment, and domestic violence sheltering. Mothers indicated that in order to save themselves, they had to sacrifice their children – something the mothers in the sample simply could not do. For example, participant 89, a White mother, said that what would have been helpful was,

If I was able to obtain drug counseling when I needed it…[but] I was the breadwinner, if I didn’t go to work, we didn’t have money. We had a daughter. If I had gotten drug counseling when I needed it, I feel I wouldn’t have ended up here. [We need to] have better support for women with children while they are getting help, like childcare.

Other mothers noted that they chose to manage their mental health and substance use disorder symptoms on their own so that they could continue caring for their children.

Mothers described how inpatient mental health and substance use disorder treatment services had no mechanism to care for women’s children and intensive outpatient services were unable to help mothers find affordable, safe childcare. Further they noted how the domestic violence sheltering system often placed age and gender restrictions on which children a woman could bring into shelter with her. Participant 166, a White mother, describes the double-bind she found herself in before coming to prison. She said, “Never enough shelters for women. Never enough transitional homes for women and their kids...In [my town] there’s a domestic violence shelter but I could only bring the baby. Going to prison is how I got free. I had to sacrifice my freedom to get free.” This theme was particularly strong as mothers discussed their attempts to access domestic violence sheltering services to escape an abusive partner. Many of the mothers we interviewed had experienced intimate partner violence in the months leading up to their incarceration, some of which was so severe that women had been hospitalized to treat their injuries. However, mothers described profound gaps in shelter access, namely that the domestic violence sheltering system was unable to ensure that they were able to escape violence with their children. As participant 11, a Native American mother, put it, “I tried to get help for domestic violence, but I couldn’t get help for being a felon. I tried to get into a shelter, but it was separate from my kids. There was no money for my girls.” Fleeing abuse meant leaving her children behind, so she stayed.

### Lack of family services during incarceration

Mothers in the study noted how the prison environment complicated their ability to successfully maintain their roles and responsibilities as mothers. Due to limitations in program availability, and the fact that some services – like residential substance use disorder treatment – were only offered in one or two prisons in the state, mothers described being forced to choose between bettering themselves and being accessible to their children. None of the prisons where we conducted interviews had a prison nursery or other intensive parenting program designed specifically to foster connection between mothers and their children. Traditional visitation was available to all incarcerated women, except for those serving their first 90 days for violating the terms of their probation in North Carolina.

Many women in North Carolina talked about the “MATCH” program – an acronym for Mothers and their Children; no similar program existed in Florida. This program – run by a non-profit organization – offers homelike visits to eligible mothers and their children at one prison in the state (Mothers and their Children - MATCH, n.d.). Participant 113, a Black mother, spoke about moving to a lower security “honor grade” facility so that she could have access to betterment programs and gain more privileges, but this move meant that she had to sacrifice her spot in MATCH. In expressing her dissatisfaction with the programs at the new facility, she said, “There’s no incentives here to make you want to do good. There is not honor grade here. No MATCH. I left ... MATCH for this. My kids were so upset.” Although personal betterment and connection with one’s children are far from mutually exclusive, the logistics of prison programs often forced mothers to choose one from among these options: participate in residential drug treatment, participate in a more intensive parenting program, or transfer to the prison closest to family to increase visits. For some mothers, choosing to participate in these programs or treatment appeared selfish to their children. These concerns will likely be amplified in the future as prisons specialize and focus all programming on one issue (e.g., mental health or substance abuse), leading more mothers to transfer between facilities to access services and programs.

Further, for some, being separated from their children catalyzed mothers to situate their lives and decisions into a broader context of their family, community, and life experiences. Many mothers described how they had to acknowledge that they were part of a potent intergenerational cycle of violence, and that they now retained very little agency in affecting outcomes for their children. As participant 98, a White mother, stated, “Now it’s a vicious cycle, my child is living in the same house dealing with the same issues because I’m here and can’t take care of him.” She was serving almost 9 years for kidnapping – a charge which stemmed from her attempt to keep her children away from their abusive father. Her distress was amplified because ultimately her own abusive parents had been granted custody.

Other mothers, however, felt empowered to break the cycle and help their children thrive. As participant 93, a Black and Native American mother, said, “Now that I’m incarcerated, I can see things for what they are, I have a choice to not repeat the cycle. I can choose to mother my kids differently so they don’t have to sit where I am now.” She was serving 90 days for conspiracy armed robbery – her first criminal charges. Mothers talked about the intersection between their behavior and their children or their roles as mothers in a variety of ways. For most of the mothers in this subsample, the mothering identity and the love they had for their children functioned as a powerful mediator in helping them to engage and sustain change processes and find new ways to connect to and mother their children.

### Mother-child connection during incarceration

Mothers described how they experienced a powerful need to create and maintain an emotional connection with their children during their incarceration. Likewise, they detailed the ways that their children, and their identity as a mother, functioned as catalysts for their change processes – whether that included leaving a violent partner, maintaining sobriety, or interrupting what they perceived as an intergenerational cycle of abuse and incarceration.

For many mothers, their children and their identity as a mother functioned as the primary source of their motivation to change. In asking for more programs to help her heal from trauma, participant 76, a White mother, simply noted, “I want to be a different kind of mom.” She was serving 38 months for felony larceny and had spent much of her life in prison. She was first arrested at age 12 for assaulting a “government official” – a truancy officer – and was incarcerated three times as a juvenile and five times as an adult for drug crimes, theft, and assault which she indicated stemmed from childhood abuse. Despite her own experiences of trauma, this participant and many others viewed their children as a source of strength and conceptualized their care and worry about their children as intrinsically motivating. As participant 154, a Black mother, noted, “I have kids to worry about – I have to be strong for them and me.” She was serving 4 years for a conspiracy robbery charge and had been in and out of prison three other times in the previous 5 years for theft. Knowing that their children were waiting for them helped many mothers cope with the psychological distress of incarceration and being separated from their children.

For many mothers in the sample, increased connection with their children fueled their desire to desist from criminalized behavior and to engage with and sustain other change processes, especially around drug use. In this way, mothers’ connection to their children and their mothering was perceived as a missed opportunity. As participant 9, a Black mother, succinctly noted, “You defeat the purpose here [of] trying to improve the lives of a mother by separating her from her kids.” Mothers described how their children’s health and well-being motivated and sustained them through the change process. Participant 2, a White mother serving 7 years for drug crimes, explained this in detail,

I love being a mother. I’m a nurturing person. I need to make up for these 7 years. It eats away at me. What mother sits here with two beautiful kids and doesn’t try to help themselves? I’ve stressed so long; all I do is stress. Just looking for love and someone to lead me or help me in the right way. Some people have that support and they take it for granted. They just don’t know how lucky they are…. I’m ready for that.

Mothers embraced, even loved, this part of their life. Further, they identified how reflecting on their children helped them make critical connections in existing in-prison programming. For example, participant 112, a White mother, spoke about the moment in a self-esteem program where she realized she did not need to remain with a violent partner. She said,

Being in here, I know now I can be happy and survive without a significant other. That’s the best thing prison did for me. Now I see myself as capable. Capable for caring for my daughters – not the best but capable. After 25 years of unhealthy relationships, I think I am choosing them [my kids].

She was serving 14 years for 2nd degree murder – she killed her partner when she discovered he was sexually abusing her children. Likewise, participant 157, a Black mother, said, “I don’t want to do drugs, I don’t want to sell them... I just want to be a better parent to my kids.” She was serving 3 years for larceny and drug possession and she had a long history of arrests and incarcerations related to drug addiction. The mothers we interviewed admitted faults and showed vulnerability as mothers. However, they consistently identified the desire to do different, and to grow and develop as women and mothers.

## Discussion

Many scholars and community activists have identified the myriad ways in which women’s needs are not being met by the criminal justice system (e.g., Bloom et al., 2003; Hoffman, Byrd, & Kightlinger, 2011; Women’s Prison Association, 2009). In Chesney-Lind & Pollock, 1995, Chesney-Lind and Pollock referred to the lack of gender-responsive policies and programs as “equality with a vengeance,” because stripping the context from the experiences and needs of men and women who make contact with the criminal justice system adds additional – and often unintentional – layers of punishment for women. It appears that little has changed in the 25 years that have passed since they made this evocative claim, and that mothering serves as an exemplar for the deep disparities experienced by incarcerated women.

Women who face incarceration experience stigma and bias from a variety of criminal justice actors (e.g., law enforcement, judges, lawyers, and juries; e.g., Tetlow, 2009). These implicit biases are typically grounded on deeply held cultural beliefs about acceptable behavior for women, and stereotypes about the types of women who become embroiled in violent relationships and engage, even tangentially, in criminalized behavior (Keitner, 2002; Snider, 2003; Wattanaporn & Holtfreter, 2014; Weare, 2013). Stereotyping and discrimination are amplified for pregnant women and mothers of young children, who are often labeled unfit, indifferent, and neglectful mothers (Aiello & McQueeney, 2016; Kauffman, 2001; Teather, Evans, & Sims, 1997). Incarcerated mothers, therefore, are subjected to additional layers of scrutiny and judgment; they are framed not simply as “criminals” or “deviants,” but as selfish, thoughtless women who made reckless decisions which did not preference their children or honor their duty as mothers (Allen et al., 2010; Berry & Eigneberg, 2003; Chesney-Lind, 2017; Moe & Ferraro, 2006). These women are often described by criminal justice stakeholders as having chosen drug use, relationships, or crime instead of choosing their children (Aiello, 2013). In this way, women’s decisions are framed as endangering the health and well-being of their children and ultimately depriving their children of having a present mother in their lives (Aiello, 2013; Cecil, 2007).

Stigma and bias were internalized by many of the incarcerated mothers we interviewed as personal shame. The mothers we interviewed sobbed while telling us stories of how they had failed themselves and their children. They were extremely distressed about the care their children were receiving during their incarceration and the loss of influence they had over their children’s lives (Easterling & Feldmeyer, 2017; Halperin & Harris, 2004). They also, however, spoke passionately about how the community had failed them and had failed their children prior to incarceration. Mothers who had tried time and again to access community resources to escape domestic violence or to enroll in substance use disorder treatment were angry that help had not been accessible. Foregoing treatment or shelter amplified their psychological distress and exposed them and their children to unnecessary adversity. For those mothers who were planning for their release from prison, they were angry that few housing programs – especially sober-living programs – existed to help them reconnect with their children while simultaneously working on their recovery.

The mothers in our sample discussed the complex, intersecting ways that mothering influenced their behavior prior to incarceration and during custody. Woven throughout these narratives was the foundational notion of wanting to do more and be more as a mother and a person, for their children and because of their children. Many of the mothers we interviewed indicated that this facet of their identity was not just absent from the prison experience, but that their ability to be mothers was actively attacked by the structures and policies of the correctional system. This phenomenon is detailed in the literature on mothering in prisons (Aiello, 2013, 2016; Luther & Gregson, 2011).

The prison system is predicated on notions of incapacitation and removal from society to protect public safety (Travis & Western, 2014). For parents, this means that part of their punishment is being physically and emotionally separated from their children. Although these policies affect both men and women, the stakes are higher – and the consequences are more severe – for mothers. Incarcerated mothers are far more likely than fathers to be the sole or custodial parent, therefore they risk having their parental rights terminated due to limitations on how long children can stay in foster care before they are “freed” for adoption (Adoption and Safe Families Act of, 1997). Mothers with no available kinship care arrangements and sentences in excess of 15 months may never be able to regain custody of their children again; in extreme cases, they may not even be given information on where their children are placed, thus effectively severing all future contact (Women in Prison Project of the Correctional Association of New York, 2006).

These policies, many of them likely well-intentioned, perpetuate the catastrophic nature of the prison experience for mothers whose needs and roles are simply not valued. There is no public outcry to defend the rights of incarcerated mothers, because, the dominant narrative is that their children would be better off without them (e.g., Allen et al., 2010). In this way, prison sentences disrupt the ability to care for, parent, and engage with one’s children, effectively enmeshing the loss of one’s status as mother as part of the punishment. Even when prisons offer more intensive parenting programs or shift the entire prison milieu to a gender-responsive and trauma-informed approach, incarcerated women are regarded as “bad mothers” (Aiello, 2016; Allen et al., 2010). The tension between rehabilitation and punishment often cannot be reconciled within these spaces, and prison staff typically default to a punishment-oriented stance (Aiello, 2013). Further, the mothering identity is rarely incorporated into other in-prison intervention programming (e.g., substance use disorder treatment or cognitive behavioral therapy-based programs designed to decrease criminal thinking) or explored as a meaningful catalyst to spark incarcerated women’s change process (e.g., Jbara, 2012; Luke, 2002).

The mothers in our sample wanted family counseling, psychological help, and emotional support both for themselves and their children. However, there were very few programs designed to facilitate basic connection between mothers and children, and restrictions and waitlists often made theses program inaccessible. Further, opportunities for family counseling in the prison setting – even for women who were planning for their release from prison – simply did not exist. Additionally, although not true of any of the facilities where data collection occurred for this project, many jail settings and some prisons do not allow physical contact between inmates and their visitors, even when those visitors are minor children (Cramer, Goff, Peterson, & Sandstrom, 2017). With a wealth of evidence suggesting that physical contact and family-friendly visiting practices increase not just child well-being, but also improve the behavior of incarcerated individuals, policies forbidding contact should be repealed and replaced.

Further, although didactic parent-education programs exist in many jails and prisons, prior research suggests that these programs fail to comprehensively address the role of incarceration on mothering and children (Aiello, 2016; Brown, 2012; Loper & Tuerk, 2006) and use mothering as a vehicle to blame and shame women for the choices or mistakes they made prior to coming to prison. In many cases, the connection with one’s children may be withheld, explicitly, as punishment for undesirable in-prison behavior (Aiello, 2013; Allen et al., 2010).

After our analyses, we were left with the sense that not using the mothering identity as a catalyst for change represented a profound missed opportunity to engage women in the intended outcomes of forensic programming: decreased in-prison behavioral infractions and decreased return to incarceration after release (e.g., Carlson, 2018; Warren, Hurt, Loper, & Chauhan, 2004; Wright, Salisbury, & Van Voorhis, 2007). Likewise, the stories told by the mothers we interviewed also suggested that the mothering identity could also be used to help support the tangential outcomes of sobriety and desistance. Thus, failing to catalyze the mothering identity as a vehicle for change represents a critical service gap as incarcerated mothers suggest that they spend a substantial amount of time in prison ruminating on the ways in which they put their children in danger and working towards growth and change in order to be better mothers to their children (Moe & Ferraro, 2006).

By focusing on the mothering identities of incarcerated women, we do not mean to perpetuate the “motherhood mystique” – the notion that women are biologically and culturally better suited to provide childcare than men, or to suggest that all women derive innate pleasure or meaning from mothering (Skott, 2016). We also do not want to reinforce the covert (or overt) message that there is one “correct” way to mother – often reflective of White, middle-class depictions of mothering (Brown, 2012; Rich, 1995; Chesney-Lind, 2006). Meaningful opportunities for women to repair, maintain, or cultivate relationships with their children, however, are conspicuously absent in prison programming. As incarcerated mothers in other samples suggest, their success at reentry is entwined with their ability to heal their families, and they indicate that conflict with their children in the days and weeks after they return home is strongly tied to relapse (Aiello, 2016).

### Strengths and limitations

A strength of the current study is that we interviewed women positioned at multiple stations within the criminal justice system, ranging from women at minimum custody serving their first 90 days for violating the terms of their probation to women at close custody serving life sentences. Mothering emerged as a theme at all three prisons and transcended variations in age, racial and ethnic identity, current charges, and sentence length. Women discussed their roles as mothers whether they were planning for release within the next few days or would spend the rest of their natural lives in prison. The sentiments shared were similar across these demographic characteristics, although women serving life sentences did not comment about services that might be helpful after their release from incarceration. They did, however, note similar needs prior to incarceration and during custody.

The current analysis should, however, be considered in terms of several limitations. First, the current study did have mothering as an eligibility criterion for participation; women were randomly selected for participation from the census at three state-level prisons. As the purpose of the research study from which data were drawn was not to examine parenting, parenting status was not collected as a demographic. Therefore, not all 187 women in the primary sample were mothers. Additionally, the prompt did not specifically ask women to reflect on their mothering identities, needs as mothers, or their children. Therefore, the themes presented are representative only of women who volunteered this information unprompted, which may indicate that they valued their identity as mothers or were in some way grappling with their mothering role and their relationship to their children. It is possible that mothers who did not volunteer this information could have divergent themes from the ones presented herein, although our analysis is reflective of others which purposively sampled incarcerated mothers (e.g., Aiello & McQueeney, 2016; Barnes & Stringer, 2014; Mignon & Ransford, 2012; Moe & Ferraro, 2006). Future research, however, should attempt to address selection bias and social desirability as factors which limit our confidence in the depth and breadth of reported results and create a more multifaceted presentation of how incarcerated women can and do mother.

### Implications for research, practice, and policy

The context of engaging in crime to provide for and protect one’s children is rarely addressed in the courtroom, and these factors do not map on to existing mitigating factors available to reduce one’s sentence length (e.g., Kennedy, Mennicke, Feely, & Tripodi, 2018; Lawrence, 2015; Spainhour & Katzenelson, 2009). Women of color often face additional discrimination and judgment as the composition of their families marks them as aberrant in the eyes of White middle-class justice system stakeholders (Richie, 2018). Policy analyses need to expand beyond investigations of gender or even the intersection of race and gender on incarcerated women’s engagement with the criminal justice system to explore the confluence of race, gender, class, and mothering (Link & Oser, 2018). It is time to challenge the inertia of a criminal justice system created by men for men based on the understanding of the needs of men which has functioned largely unchanged for a century. A first step might be integrating evidence-based and gender-responsive risk-needs assessment (e.g., Van Voorhis, Salisbury, Wright, & Bauman, 2008) to gain a comprehensive understanding of mothers’ needs and develop policies and programs which explicitly address these needs.

Likewise, the impact of prison specialization on incarcerated women and their children demands analysis. With few women’s prisons in most states, mandating women with mental health or substance use disorders to be incarcerated in the one facility with relevant programs likely increases her distance from her home and her children. Several of the mothers we interviewed noted how they had to choose between entering treatment and receiving visits from their children.

Like other samples of incarcerated mothers (e.g., Ferraro & Moe, 2003; Hunter & Greer, 2011; Parry, 2018), the mothers we interviewed positioned their criminalized behavior in the context of caring for and protecting their children. Ferraro and Moe (2003) noted that the decision to engage in criminalized behavior was situated in the context of economic need by incarcerated women – women described stealing goods or passing worthless checks as a means to feed themselves and their children. The vast majority of those living in poverty in our nation are head-of-household women with minor children who are responsible for meeting the financial and emotional demands of their family (Fontenot, Semega, & Kollar, 2018). Therefore, criminalized behavior is often entangled with the lack of health insurance and childcare, and the difficulty of weighing the cost of childcare against the potential salary of low-wage jobs (Ferraro & Moe, 2003). Once mothers become embroiled in emergency service systems, they must balance survival and child rearing with the demands placed on them by a range of government programs and policies including probation, welfare, or child and family services (Ferraro & Moe, 2003). For some mothers, engaging in nonviolent crime like theft or fraud was perceived as a reasonable vehicle to ensure the survival of their children without directly harming other people (Ferraro & Moe, 2003).

Prior research suggests that low-income mothers are far less likely than their middle-class counterparts to engage in substance use disorder treatment due to lack of child care; these gaps are amplified for women who have two or more children, children younger than five, and women of color (Rosen, Tolman, & Warner, 2004). However, few communities have established mother-child residential treatment programs, where mothers receive substance use services and children are both incorporated into their mother’s recovery and receive their own therapeutic services (e.g., Seay, Iachini, Dehart, Browne, & Clone, 2017). Likewise, the domestic violence sheltering system is perpetually under-resourced, turning away thousands of requests for help across the nation every day (National Network to End Domestic Violence, 2016). The access gap to these services is inextricably entwined with the criminal justice system as mothers attempt to survive and cope with their situations. Many of the mothers we interviewed suggested that they tried to escape from lives characterized by violence, addiction, or crime, but were ultimately trapped inside these circumstances as they could not escape with their children. This sentiment was strongest among women who indicated that after failing to access community-based substance use or sheltering programs, they returned to a problematic (typically male) intimate partner and were subsequently prosecuted as a co-conspirator or accomplice to his crimes.

And finally, child welfare policy and procedure require comprehensive reform to facilitate parenting from prison. Incarcerated women are serving prison sentences as punishment for their crimes; the loss of physical contact with and parental rights to their children should not be part of that punishment. As noted, the vast majority of research on incarcerated mothers focuses on their children, and incarceration is associated with a range of negative behavioral, emotional, and justice-system outcomes for those children. Therefore, the policies which keep children from their mother during her incarceration or terminate her parental rights as a function of that incarceration, need to be examined in the context of the health and wellness of those children. If both the criminal justice and child welfare systems could identify ways to promote safety while increasing connection, love, visitation, education, and mothering, outcomes among mothers and children would likely be improved.

## Conclusion

The current secondary data analysis explored experiences of mothering before and during incarceration and examined how mothering intersected with incarcerated women’s health and health outcomes. The mothers in our sample detailed having sacrificed their own health and wellness in order to parent their children. Many described foregoing substance use disorder treatment because they were unable to bring their children or identify suitable childcare. Mothers also described the psychological distress of family separation. They were eager to participate in parenting programs designed to increase mother-child connection and facilitate visits and they identified the mothering role as a key mechanism of change in substance use disorder treatment programs. The prison environment offers few opportunities for mothers to connect with their children; most mothers never receive even one visit from their children during incarceration. Recommendations include infusing mothering and caretaking responsibilities into the sentencing process and exploring the intersection of race, gender, class, and mothering status on criminalized behavior. Additionally, there is an urgent need to expand the availability of residential community-based substance use disorder treatment programs that allow women to receive treatment and mother their children. Intensive parenting programs that facilitate connection between mothers and children during incarceration are also urgently needed.

## Data Availability

The datasets analyzed during the current study are available from the corresponding author on reasonable request.
